# Transmission of bovine H5N1 virus in a hamster model

**DOI:** 10.1128/jvi.00147-25

**Published:** 2025-03-25

**Authors:** Kiyoko Iwatsuki-Horimoto, Ryuta Uraki, Mutsumi Ito, Tong Wang, Lizheng Guan, Peter Halfmann, Amie Eisfeld, Gabriele Neumann, Seiya Yamayoshi, Yoshihiro Kawaoka

**Affiliations:** 1 Division of Virology, Institute of Medical Science, University of Tokyo 26430 , Minato-ku, Tokyo, Japan; 2 The University of Tokyo Pandemic Preparedness Infection and Advanced Research Center (UTOPIA), University of Tokyo, Minato-ku, Tokyo, Japan; 3 The Research Center for Global Viral Diseases, National Center for Global Health and Medicine Research Institute, Shinjuku-ku, Tokyo, Japan; 4 Influenza Research Institute, Department of Pathobiological Sciences, School of Veterinary Medicine, University of Wisconsin-Madison 70738 , Madison, Wisconsin, USA; 5 Department of Special Pathogens, International Research Center for Infectious Diseases, Institute of Medical Science, University of Tokyo 26430 , Minato-ku, Tokyo, Japan; St. Jude Children's Research Hospital, Memphis, Tennessee, USA

**Keywords:** influenza virus, bovine H5N1, transmission, hamster model

## LETTER

**Fig 1:**
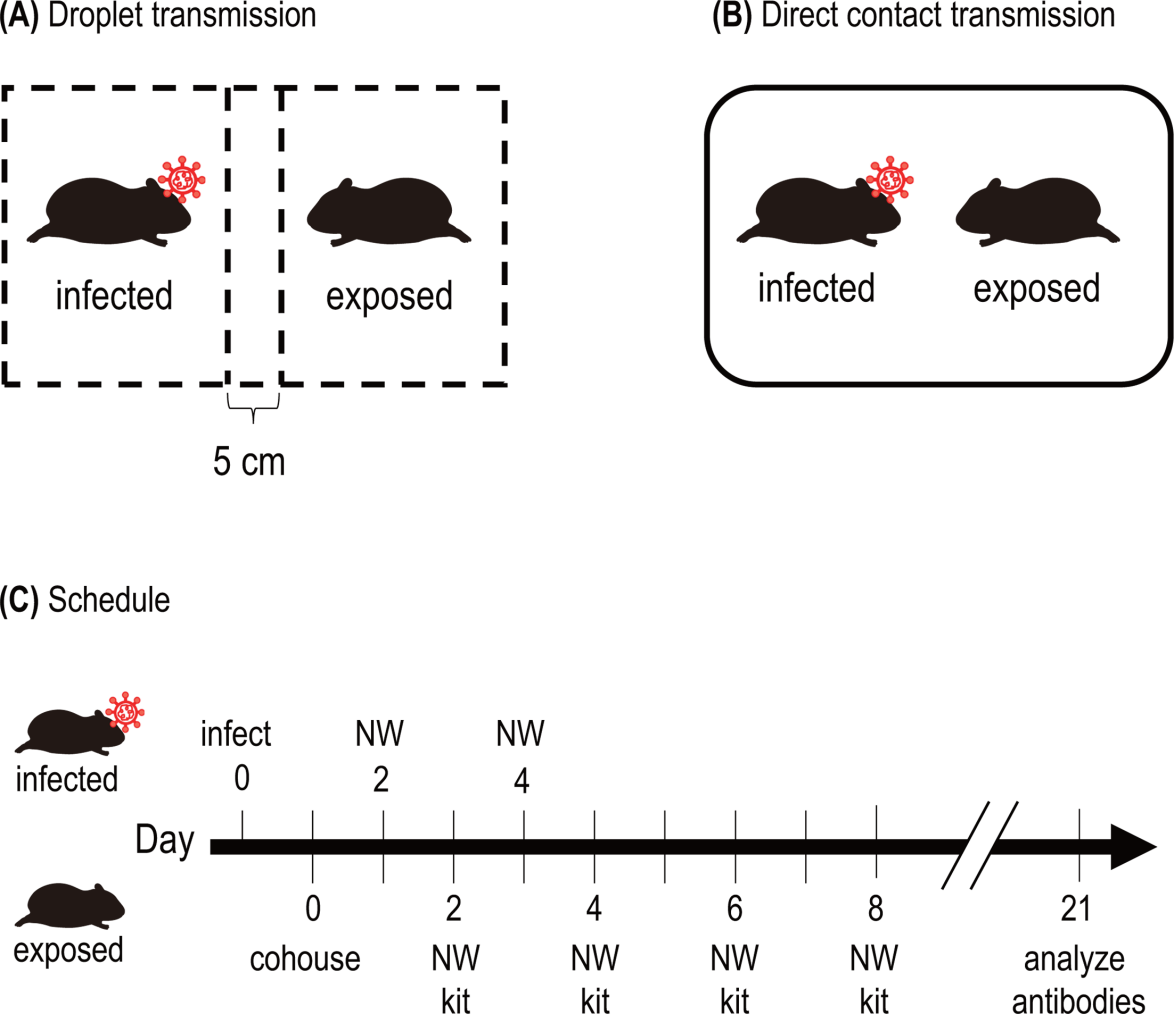
Schematic representation and schedule of the transmission study. (**A and B**) Schematic representation of the droplet (**A**) and direct (**B**) contact transmission cages. Droplet transmission cages have two wire-mesh partitions that prevent direct and indirect contact between the animals but allow the spread of the virus through the air. The cage is made of wire to facilitate airflow (A; shown as dashed lines). An infected hamster was placed in a chamber of the droplet transmission cage (**A**) or direct contact cage (**B**). Twenty-four hours after infection, one naïve hamster was placed in the adjacent chamber of the droplet transmission cage (**A**) or the same direct contact cage (**B**). (**C**) Schedule of the study. NWs were collected every other day from the infected (C, upper row) and exposed (C, lower row) animals for virus titration. Virus titers were determined by performing plaque assays on Madin-Darby canine kidney cells. The NW of the exposed hamsters was also tested with a rapid diagnostic kit, and if positive, hamsters were euthanized, and their nasal turbinates, lungs, and brains were collected for virus titration. Serum was collected from the remaining hamsters on day 21 post-exposure and analyzed for seroconversion by using a hemagglutination inhibition assay.

Transmission among mammals of bovine highly pathogenic avian influenza (HPAI) H5N1 viruses, which have caused outbreaks in US dairy cattle (1–3), has been demonstrated in ferrets by our group (4, 5) and the US Centers for Disease Control and Prevention (CDC) (6). These studies showed that these viruses can be transmitted among ferrets via respiratory droplets, albeit with lower efficiency than seasonal human influenza viruses. In contrast, bovine HPAI H5N1 viruses spread easily among ferrets through direct contact (3 of 3 [100%] ferrets) (6). Although ferrets are frequently used for influenza virus transmission (7–9) and vaccine efficacy (10, 11) studies, they demand considerable housing space and personnel and can be difficult to handle. Here, we investigated the transmissibility of the bovine HPAI H5N1 virus A/Texas/37/2024 (TX/37), which was 100% lethal in ferrets inoculated with as little as 10 plaque-forming units (PFUs) (5) by using a hamster model. Six-week-old Syrian hamsters were anesthetized and intranasally infected with 10^3^ PFU of TX/37. To study respiratory droplet transmission, we placed an infected animal in one chamber of a transmission cage that had two wire mesh partitions 5 cm apart to prevent direct and indirect contact between animals (Fig. 1A) and placed a naïve hamster in the other chamber in 24 h post-infection (four pairs). To study direct contact transmission, we cohoused an infected hamster with a naïve hamster (Fig. 1B) at 24 h post-infection (five pairs). Nasal washes (NWs) with 400 µL of phosphate-buffered saline (PBS) were collected every other day starting on day 2 post-infection or post-exposure (Fig. 1C), and virus titers were quantified by performing plaque assays in Madin-Darby canine kidney cells. The NW of the exposed hamsters was also tested by using a rapid diagnostic kit (ESPLINE Influenza A&B-N, FUJIREBIO, Japan), and if positive, the hamsters were euthanized, and their nasal turbinates, lungs, and brains were collected for virus titration.

**Fig 2:**
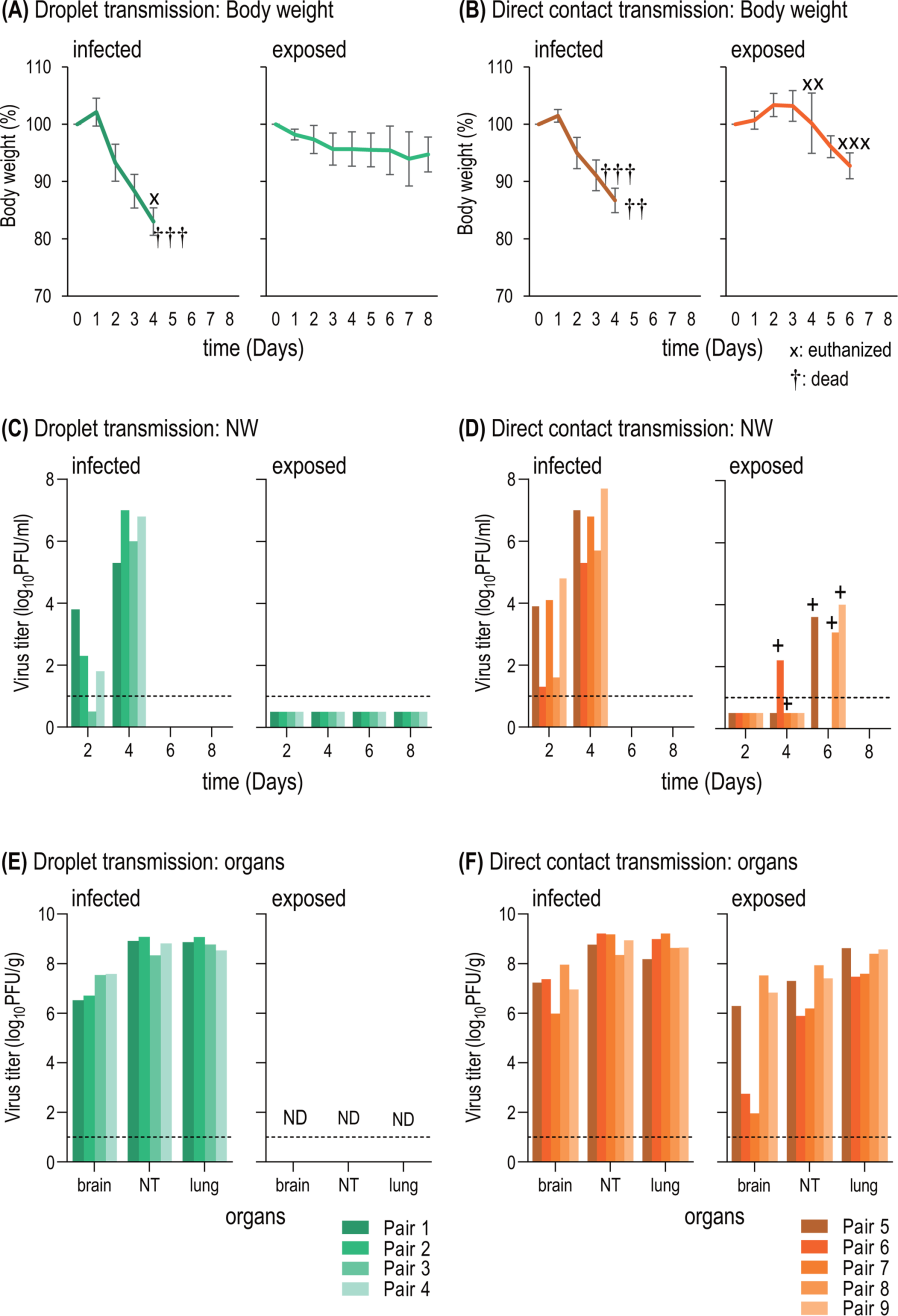
TX/37 is lethal in hamsters and spreads by direct contact but not respiratory droplets. Hamsters were anesthetized with isoflurane and intranasally inoculated with 10^3^ PFU of TX/37 in 30 µL of PBS. (**A and B**) Body weight changes. Body weights were monitored daily. Baseline body weights were measured prior to infection or cohousing. Data are means ± SEM. x: euthanized, †: dead. (**C and D**) Virus titers in NW. NW was collected every other day starting on day 2 post-infection or post-exposure and was quantified by performing plaque assays. The NW of the exposed hamsters was also tested by using a rapid diagnostic kit on the same day as the sample collection. +: Rapid diagnostic kit positive. The lower limit of detection is indicated by the horizontal dashed line. (**E and F**) Virus titers in organs. Virus titers in the nasal turbinate (NT), lung, and brain tissues of dead or euthanized hamsters were quantified by performing plaque assays. ND: not done.

Infected hamsters exhibited weight changes (Fig. 2A and B, left panels), with one hamster being humanely euthanized because it lost more than 20% of its body weight (Fig. 2A, x in the left panel); the other hamsters died before their body weights decreased by 20% by day 5 post-infection (Fig. 2A and B, † in the left panels). A virus was detected in nasal swabs of all infected hamsters on days 2 and 4 post-infection (Fig. 2C and D; left panels) and spread to respiratory organs and brains of all animals (Fig. 2E and F, left panels). All direct contact exposed hamsters exhibited weight changes (Fig. 2B, right panel) and were positive based on the rapid diagnostic kit by day 6 post-exposure (Fig. 2D, + in the right panel). Although the virus titer in the NW of one hamster (pair 7) was below the detection limit despite being positive in the rapid diagnostic kit (Fig. 2D, right panel), the virus spread to the nasal turbinates, lungs, and brains of all directly exposed hamsters (Fig. 2F, right panel). In contrast, there was no significant weight change (Fig. 2A, right panel), no virus was detected in the NW (Fig. 2C, right panel) of the droplet-exposed hamsters, and none had seroconverted by day 21 post-exposure (data not shown). Bovine HPAI H5 virus was thus found to be highly pathogenic and highly transmissible by direct contact in hamsters, although we did not detect respiratory droplet transmission. Therefore, hamsters have potential as a small animal model for analyzing the protective effect of vaccines or antiviral drugs against bovine HPAI H5 virus infection.
